# Chimpanzees attribute agency to virtual animals, but not to self-propelled shapes

**DOI:** 10.1098/rspb.2026.1574

**Published:** 2026-09-09

**Authors:** Emilie Rapport Munro, Matthias Allritz, Ken Schweller, Francine Dolins, Daniel B. M. Haun, Josep Call

**Affiliations:** ^1^ Department of Comparative Cultural Psychology, Max Planck Institute for Evolutionary Anthropology, Leipzig, Sachsen, Germany; ^2^ Ape Cognition and Conservation Initiative, USA; ^3^ University of Michigan-Dearborn, Dearborn, MI, USA; ^4^ School of Psychology and Neuroscience, University of St Andrews, St Andrews, Fife, UK

**Keywords:** virtual environments, agency, chimpanzee, social cognition

## Abstract

Discriminating agents from inanimate objects is essential for predicting others’ behaviour. Although chimpanzees (*Pan troglodytes*) differentiate between agents and objects, the cues underlying this ability remain unclear. We investigated chimpanzees’ agency attribution using a virtual-environment paradigm in which they interacted with four competitors varying in two putative agency cues: self-propulsion and animal-like morphology. We measured facial, vocal and gestural emotive responses directed at these competitors as behavioural markers of agency attribution. Chimpanzees showed clear evidence of attributing agency. The effect of self-propulsion was not significant, but there was a strong effect of morphology: subjects responded more strongly to animal-shaped virtual competitors than to geometric shapes. This pattern resembles that shown by human infants and adults: they, too, identify entities with animal features as agents, but self-propelled movement without cues of internal control is not enough to denote agency for these populations. These converging results point to a shared, morphology-based mechanism guiding agentive responses in chimpanzees and humans.

## 1. Introduction

Agents are entities that possess a set of characteristics distinguishing them from mere physical objects. Leslie's [1] ‘tripartite theory of agency’ specified these characteristics in three classes:

— Mechanical properties: agents possess an internal source of energy via which they can act on the world around them.
— Actional properties: agents’ actions are in pursuit of goals (they are intentional). They are perceptive and reactive to changes in their environment. They can interact with other agents.
— Attitudinal or cognitive properties: agents’ actions, reactions and interactions are determined by relationships with information.

It has been proposed that humans make use of these distinct properties to attribute agency, that is, to differentiate between agents and physical objects, and to make rapid judgments about the agency of a novel entity upon first encountering it [1]. Before going further, it is important that we make one point clear: following Leslie [1,2] and others (e.g. [3]), we draw a distinction between agents and animate objects. Although most or all agents are indeed animate, animacy is considered to be *external to the concept of agency*: not all animate objects are necessarily agents. Therefore, when we discuss agents throughout this work, we refer to entities that possess, and are represented as possessing, mental states—as described in the definition above.

The ability to attribute agency is important and useful to humans for several reasons. First, relevance: since conspecifics, prey and predators—highly salient entities with which we need to interact—are all represented by the category of ‘agents’, prioritizing information relating to this category is a crucial tool [4,5]. Second, opportunities: agents afford a different set of interactions in contrast to non-agentive objects (e.g. communication, conflict and cooperation), and only when interacting with agents are reciprocal and context-dependent behaviours relevant. Third, prediction: since agents are intentional, attributing agency to another entity allows us to predict and explain the behaviour of that entity in terms of goals [6].

Extensive research has been dedicated to investigating how human infants understand agency (for an overview, see [7]). Some authors argue that human infants are born with a skeletal version of a psychological reasoning framework that enables them from the age of only a few months to (i) identify agents, (ii) reason about those agents’ actions by inferring the mental states they are based on and (iii) predict those agents’ actions [7]. We will return to this putative framework in more detail in the discussion section of this paper.

The above arguments could also apply to other animal species, particularly those living in social groups, to explain why they might also benefit from an evolved ability to discriminate intentional agents from non-agents and to interact with them accordingly. Despite this, very little experimental or theoretical work has been dedicated to the question of whether and how non-human animals attribute agency. Although some work hinges upon certain animal species treating others as agents—for instance, studies considering primates’, corvids’ and dogs’ understanding of others’ visual perspectives, goals, desires and beliefs (see e.g. [8–14])—rarely have scientists asked the question of how these animals determine *which entities* are endowed with such perspectives, goals and beliefs to begin with. Chimpanzees are an obvious study species in this respect, for reasons that will be detailed below.

### (a) Chimpanzees’ attribution of agency

Chimpanzees (*Pan troglodytes*) are a species characterized by complex sociality. In the wild, they generally live in large communities—varying from a couple of dozen individuals to over a hundred [15–17]—governed by dominance hierarchies and patterns of coalition and alliances. Their fission-fusion grouping system, in which the community splits into subgroups for feeding, travelling and sleeping, adds an additional level of complexity, lending fluidity to the size and character of their parties [18]. They frequently compete for access to food and mates, but also engage in affiliative and cooperative behaviours including grooming, group hunting and territorial patrols [18].

Several aspects of chimpanzees’ rich social behaviour, such as reciprocal exchange [19], alliance-building [18] and audience-specific communication [20,21], strongly suggest that they attribute agency. Experimental results provide further evidence: chimpanzees adjust their behaviour based on what others can see and hear [8,9,22,23]. They choose correct solutions to human problems [24] and help others according to their intentions [14,25]. Most informatively, chimpanzees distinguish between accidental and intentional actions [26], distinguish partners who are unwilling from those who are unable to complete an action [27] and differentially punish those individuals who deliberately stole their food from those who did not [28].

Further evidence shows they distinguish agents from non-agents: Kano & Call [29] presented chimpanzees with videos of a human hand or a mechanical claw grasping one of two objects. After the target locations were switched, the hand/claw paused midway between objects. Eye-tracking revealed that chimpanzees expected the hand, but not the claw, to pursue the same target as before. Since both completed identical actions, this indicates that chimpanzees treated the hand as an agent with goals, and the claw as a non-agentive object.

What is not yet clear is what particular cues chimpanzees use to distinguish agents from physical objects. Do they operate with specific rules or biases, based on perceptual features or behaviours, that they use to spontaneously judge the agentiveness of objects that they encounter for the first time? [29] only provided information on how chimpanzees attribute agency to entities that differ in morphological characteristics but move in the same way. What about when entities differ in their behaviour? Also, although we have seen that captive chimpanzees discriminate between a human hand and a plastic claw, hands are appendages with which the subjects were already familiar; they might have learned over their lifetime that an entity sporting such an appendage was an agent, rather than using the hand's features to sort it into a category of agents. What about when they come across entirely novel entities?

### (b) The current study

In a recent experiment [30], chimpanzees in a virtual environment (VE)-based paradigm interacted with novel virtual competitors (animated elephants and gorillas). We observed that when they lost rewards to the virtual competitors, six chimpanzees exhibited atypical behaviours, such as vocalizing, spitting and banging on the competitor's image. We suspected that these reactions were not simply an indicator of their frustration (since these subjects had extensive experience with losing at trials in virtual environment experiments but had rarely, if ever, produced comparable responses) but postulated that the chimpanzees were categorizing the virtual competitors as goal-directed agents with which they were attempting to remonstrate. The virtual competitors in that study possessed two possible indicators of agency: they were self-propelled and had animal-like morphology.

These outcomes raised several questions: are both of the above cues required for chimpanzees to identify a novel entity as an agent? Or would either animal features or self-propulsion individually be sufficient?

We aimed to answer these questions in the current study. The same six chimpanzees as in [30] interacted with novel entities that varied along two axes: self-propulsion and the presence of animal features. We compared the frequency with which chimpanzees responded emotively towards these competitors as a measure of relative levels of agency attribution.

We hypothesized that chimpanzees would interpret some or all of the virtual competitors that possessed at least one agency cue as agents, and that this classification would be apparent in the chimpanzees’ emotional reactions towards these competitors when the competitors ‘stole’ virtual prey from them. We predicted that our subjects would show negligible responses towards the stationary shape competitors (which lacked any indicators of agency) but would show greater responsiveness to at least some of the other competitors. However, we did not have specific predictions about which of the self-propulsion or animal-like features would engender greater responses, or whether they would be equally effective.

Our aim was also to generate systematic and quantifiable evidence for chimpanzees attributing agency to virtual entities. The use of virtual environments to study chimpanzee cognition is a very new paradigm, and much remains unknown about how chimpanzees interact with VEs. A finding that chimpanzees spontaneously assign agency to novel virtual entities would be very interesting and would lend weight to the contention that the use of VEs is valid in testing chimpanzees’ sociocognitive skills.

## 2. Methods

### (a) Subjects and housing

Six chimpanzees (four females, two males, mean age = 25.2 years, s.d. = 18.4 years) housed at the Wolfgang Köhler Primate Research Center (WKPRC) in Leipzig Zoo, Germany, participated in this study. All subjects had prior experience of navigating three-dimensional virtual environments via the touchscreen, interacting with virtual competitors and collecting virtual prey items [30]. For descriptions of the virtual training methods and history of the Leipzig apes, see [31,32]. Subjects participated in the study voluntarily by entering the research room adjoining their main indoor enclosure.

### (b) Materials

A 27-inch clear infrared touchscreen was installed in the research room, in the wall separating the chimpanzee section from the experimenter section. A full-colour monitor was mounted behind the touchscreen, with the monitor's visual feed displayed through the touchscreen. The monitor and touchscreen were connected to a laptop controlled by the experimenter; any touches made on the screen were relayed to the laptop. Audio feedback was played through a pair of speakers also connected to the laptop. The subjects and the touchscreen were filmed by a camcorder on a tripod; the experimenter (E.R.M.) viewed the live feed on a tablet. The software used was APExplorer3D, programmed in C# using Unity3D. The program showed a virtual environment from the perspective of an invisible agent, whose movement subjects could control by touching the screen. Touches to anywhere in the environment other than the sky caused the invisible agent to ‘walk’ towards that object/location, until it reached the specified location, or it contacted an impermeable obstacle or another touch was recorded. Touches made to the far left- and right-sides of the screen caused the agent to rotate in that direction while also moving towards the touched location.

#### (i) Stimuli

**Figure 1.**
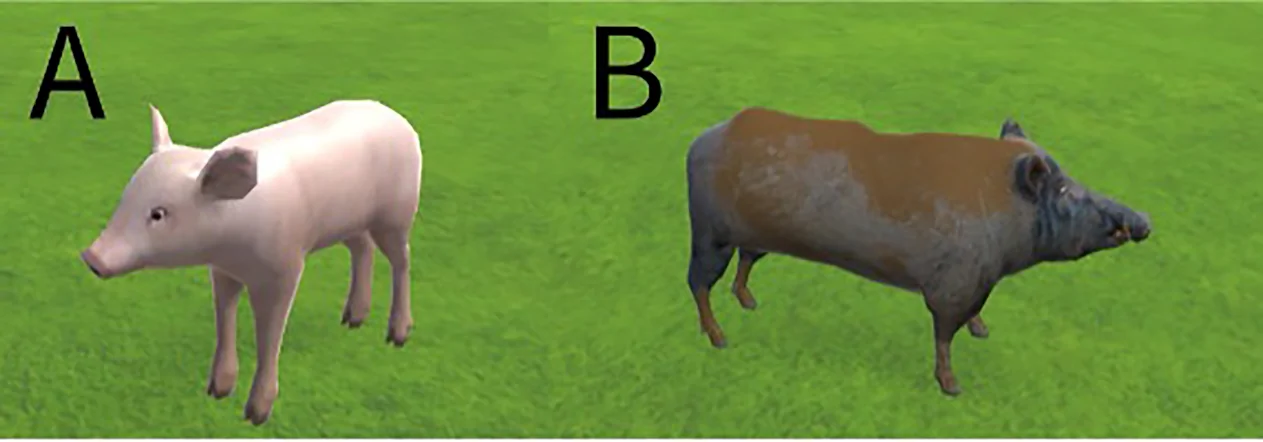
The virtual prey. (A) Pig prey. (B) Boar prey.

The targets were two virtual prey animals: a pig and a boar (figure 1).

Subjects were required to direct the invisible player to ‘walk’ towards and ‘collide’ with one of the virtual prey. When the invisible player made contact with a prey item, a ‘ta-daah’ sound played, and the prey fell over and lay motionless on the ground for 2 s before disappearing. The subject was then rewarded with fruit by the experimenter (see below).

**Figure 2.**
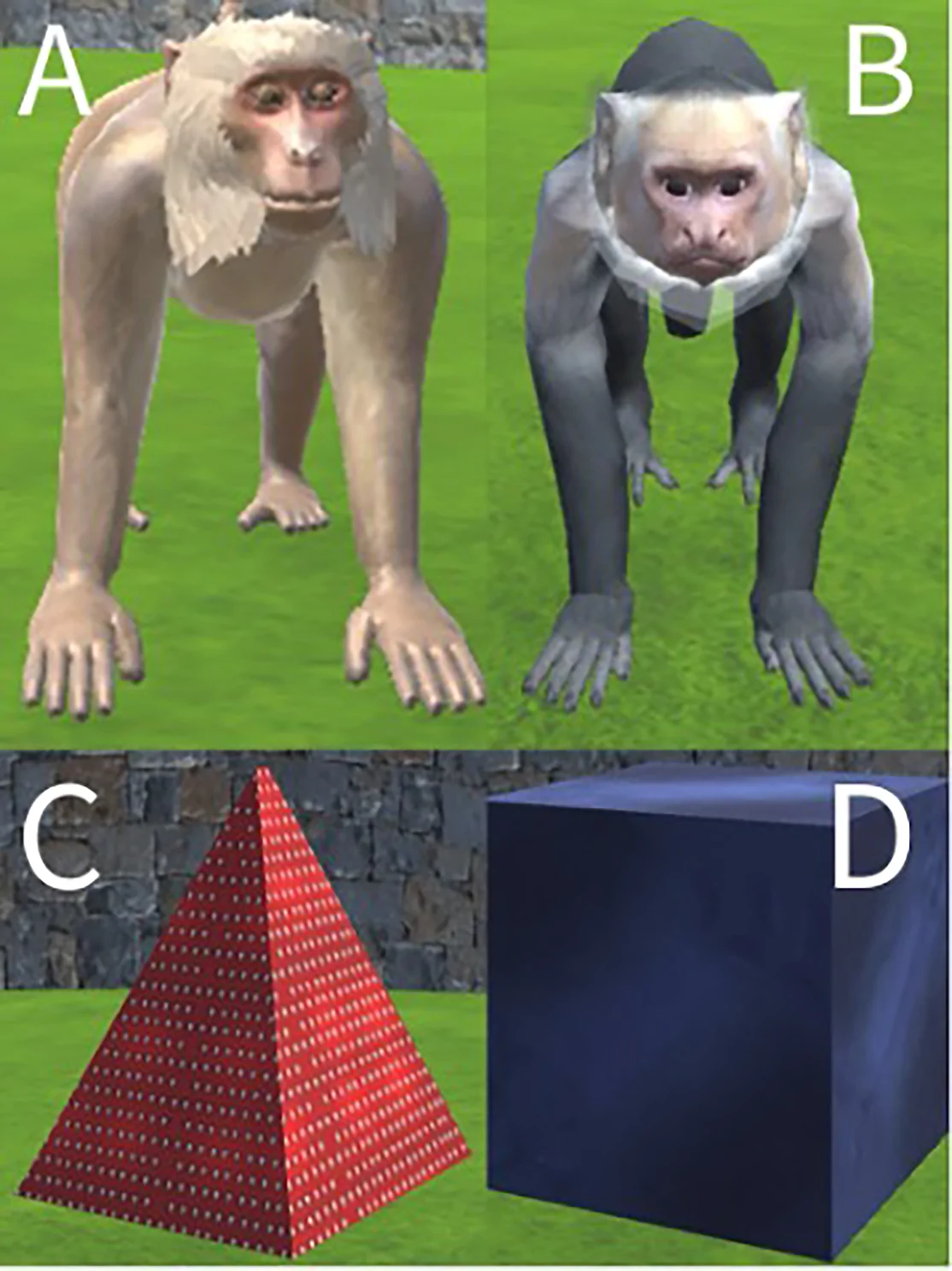
The virtual competitors. (A) rhesus macaque; (B) capuchin; (C) red pyramid; (D) blue cube.

There were four virtual competitors that differed along two axes: features and movement. Two competitors were designed to resemble animals—a capuchin and a rhesus macaque—and two were geometric coloured shapes: a blue cube and a red pyramid (figure 2). One competitor of each feature type was stationary, while the other was self-propelled and could move through the virtual environment along a fixed path towards the virtual prey. Which competitor was assigned to which movement type was counterbalanced between subjects. For example, for subject Azibo, the rhesus macaque and the red pyramid were the moving competitors, and the capuchin and the blue cube were stationary. The shapes moved through the environment by gliding, while the monkeys walked using their limbs. The heads of both animal competitors, whether they were assigned to the stationary or self-propelled condition, bobbed slightly in a repetitive manner; this was not counted as self-propulsion because the movement was very small and was continuous from the start of the trial, and therefore not obviously self-initiated.

#### (ii) Rewards

Every time a subject successfully collected a virtual prey item, they were rewarded with two small pieces of fruit: a chunk of apple *and either* half a grape or a slice of banana (alternating, because there are limits on how many pieces of these latter fruits the chimpanzees are allowed each day).

### (c) Procedure

We administered three types of trials: baseline, test and motivation.

#### (i) Baseline

All subjects had experience with traversing virtual environments to collect virtual prey. Specifically, they had each participated in a stepwise training procedure designed to teach them how to direct their player's movement in the virtual world (i.e. by touching the screen where they wanted their player to go) and that when their invisible player collided with a virtual target, they would be rewarded with food [32]. Two adult participants had also completed a study in which they learned to use landmarks to navigate and locate targets in a large, semi-naturalistic virtual environment [31]. Finally, all subjects had participated in a study in which they competed with virtual, moving animals (an elephant and a gorilla) for access to moving virtual prey (a chicken, a dog, a pig and an antelope) [30].

However, it was important to ensure that the chimpanzees remembered how to collect the virtual prey and understood that doing so would result in a food reward prior to the start of testing. We therefore began with a short baseline procedure. Sessions consisted of trials featuring one or both prey items, with nothing else present in the arena; subjects simply had to cause their invisible player to walk up to and collide with the prey to receive a fruit reward. Each session was made up of 18 trials: six trials with each prey individually, and six trials with both the pig and the boar together. Subjects received a minimum of two sessions of this training and a maximum of 10; the criterion for moving on to the test was that an individual must succeed in collecting all prey items in all trials in a single session. No subjects required more than the minimum of two training sessions.

#### (ii) Test trials

We conducted two types of trials in which the subjects couldn't win. In these trials, they received no fruit reward. In *moving-competitor* trials, the subject was located in the centre of the arena, facing the prey animal, which was a few metres away to the left or right. The moving competitor began behind the prey, closer to it than the subject was. There was an invisible trigger line a short distance in front of the subject's starting point. This meant that when the subject player began walking towards the prey, the competitor was likewise triggered to move. The competitors began the trials already facing the prey and always took fixed straight-line paths towards it. Because the competitor began closer to the prey than the subject was, the competitor always got there first. When the competitor made contact with the prey, which occurred about 5 s after the beginning of the trial, a reward chime played, which was distinct from the ‘ta-daah’ sound that played when the player controlled by the subject collected the prey. The prey then fell over and lay motionless on the ground for 2 s before disappearing. Each trial lasted 30 s, so once the competitor had collected the prey, the trial continued until the time elapsed, allowing us to record the subject's responses to the onscreen competitor.

In *stationary-competitor* trials, the starting positions of the subject and prey were the same as in moving competitor trials, but the competitor stood right beside the prey, again facing it directly. 5 s after the trial began, the prey fell over and lay motionless for a further 2 s before disappearing (this timing was chosen to match the timing of the prey falling over in the moving-competitor trials). The subject was restrained from ‘walking’ through the arena for the first 2 s of the trial, meaning that they couldn't reach the prey before it fell over and became inaccessible. The trial lasted for 20 s in total, meaning that after the prey fell over, there were further 15 s in which the subject could respond to the competitor. Although the competitor, being stationary, did not actively ‘collect’ the prey by colliding with it, we were confident that the competitor's proximity to the prey would be sufficient for the subjects to attribute responsibility for the prey's disappearance to the competitor, *if* the subjects viewed the competitor as an agent.

#### (iii) Motivation trials

It was important that subjects did not lose to the competitors in *every* trial. To maintain subjects’ motivation, we included two different prey types: each competitor had one prey they ‘liked’, and one prey that they ‘disliked’. The goal was to mitigate the potential agitation caused by losing repeatedly, avoid learned helplessness, and also mitigate the possibility that subjects’ responses might carry over from the end of one trial to the beginning of the next, leading to an overestimation of responses in trials that follow those that strongly elicit responding. For each subject, the boar was assigned as the ‘liked’ prey for two competitors, and the pig was ‘liked’ by the other two competitors. This assignation was counterbalanced across individual subjects. Trials featuring a competitor's ‘liked’ prey ran as described above: the competitor competed with the subject for access to the prey. In trials featuring a competitor's ‘disliked’ prey (i.e. motivation trials), the competitor, instead of competing, ignored the prey, leaving the subject free to collect it. We had two types of motivation trials depending on the competitors’ behaviour. With the self-propelled competitors, the starting positions of the competitor, the prey and the subject were the same as in test trials. Three seconds after the trial began (regardless of what the subject did), the competitor began moving in a straight line *away* from the prey. After the competitor had moved a short distance, it stopped and remained stationary for the rest of the trial. The subject was therefore free to approach and collect the prey. As in the test trials, the competitors’ paths were fixed and invariable. In motivation trials featuring the stationary competitors, they were positioned directly next to the prey item as in test trials; however, the prey did not fall over and disappear unless the subject collided with it.

#### (iv) Sessions

Following the two training sessions, each subject completed 20 test sessions. A test session comprised 24 trials: 12 test trials and 12 motivation trials. Subjects were therefore guaranteed a fruit reward in 50% of trials within a session; they completed one session per testing day. Each of the four competitor types (moving animal, stationary animal, moving shape and stationary shape) was presented in mini-blocks of three test trials. This was done to reduce carryover of emotional responses from trials with one competitor type to those with another. To avoid a predictable trial order and maintain motivation levels, three motivation trials were interspersed within the mini-blocks of three test trials in a random order. See table S1 in the supplementary text for an example session. Thanks to this system, trials with the potential to be confounded by carryover, while present, were very rare, namely 0.42% of all trials.

Prior to data collection, we completed a power analysis via data simulation. Based on best guesses about relationships between variables and effect sizes, we generated artificial datasets to which we then fitted binomial models, and, in a final step, estimated how often these models would yield a significant result. We found that testing a subject with 240 trials would give us enough power to detect an existing effect in both predictors in 80.1% of simulations. This informed our decision to give subjects 20 sessions with 12 test trials per session. Please consult the supplementary text for more information on the power analysis.

### (d) Data coding

Our dependent variable was the frequency with which subjects responded emotionally to the different competitors when they lost out on virtual prey and their associated rewards. Responses were coded from the videos made during the sessions. E.R.M. watched each video and recorded, for each test trial, whether or not a response was observed. A second coder, who was blind to the study's research questions, watched a randomly selected subset of 20% of the videos and coded them using the same ethogram. The interrater reliability test gave a Cohen's kappa of 0.76, indicating substantial agreement. E.R.M.'s coding was used for all analyses.

Our unit of measurement was binary: for each test trial, we were interested in whether each subject showed *any* emotional response to the competitor. Every trial's response was therefore designated as 1 or 0. In many cases, subjects made several different responses within a single test trial, but this was not treated differently from trials in which only one response was recorded. We decided on this method so as to avoid potential complications in defining a unit of response and in distinguishing between a single response and multiple responses. See table 1 for the full ethogram.

**Table 1.** Ethogram.

reaction type	subsets	characteristics	behaviours not counted	possible function
vocalization	cough	low, quiet, short and usually unvoiced sound	vocalization made in response to other vocalizations in the background vocalization made while subject is looking away from screen vocalization made while competitor not visible onscreen	indicator of annoyance, mild threat given by dominant individual to subordinate often accompanied by threat gestures, e.g. ground slaps and arm raises [18,33]
facial expression	pout	lips extended but close together, as if subject whistling	expression made while competitor not visible onscreen or while subject looking away from screen	indicator of distress [18]
	open/close mouth	rapidly open and shut mouth as if vocalizing but without sound	expression made while competitor not visible onscreen or while subject looking away from screen	
gesture	spit	suddenly bring face close to screen with sound of air being expelled from lips	gesture made while competitor not visible onscreen or while subject looking away from screen	attention-getting [34]
	head shake	move head up and down or rotate head back and forth from shoulder to shoulder while looking at competitor.	gesture made while competitor not visible onscreen or while subject looking away from screen subject frequently bobs/moves head throughout trials, and the head shakes if question is not distinct, i.e. different from subject's general moving patterns	in chimpanzees, head shaking serves a preventative function as in humans [35] and is used in agonistic contexts [36]
bang	knuckles	use knuckles to touch the competitor, making an audible sound	touch in question is not obviously harder (makes louder sound) than other touches within the same trial, i.e. if construable as simply trying to walk into/through/past competitor touch is made when competitor is not onscreen or while subject is looking away from screen competitor is onscreen but the location touched is >3 cm away from competitor in any direction	drumming displays serve intimidatory purposes [18]; pokes and slaps used in agonistic contexts [36]
	slap	use palm or back of open hand(s) to touch competitor	if subject frequently uses palm/back of hand/fist to touch screen in general movements, and this touch is not obviously harder than usual touches if touch is made when competitor is not onscreen or while subject is looking away from screen if competitor is onscreen but the location touched is >3 cm away from competitor in any direction	
	fist	use closed fist(s) to touch competitor		
press	hand	use one or two hands to press (distinct from banging) hard on the screen; causes screen to make a cracking sound	reaction made while competitor not visible onscreen or while subject looking away from screen	
	back foot	use back foot to touch competitor on screen.	reaction made while competitor not visible onscreen	
jump		jump up and down in front of screen; usually accompanied by a vocalization	walking away from/to side of the screen and then jumping reaction made while competitor not visible onscreen or while subject looking away from screen	jumps sometimes occur in drumming displays as intimidation tactic [18]

Only responses that were clearly directed towards a competitor were recorded. For example, a grunt would not be recorded if it was made when the competitor was not visible onscreen, or when the subject was looking away from the screen. Another type of response was subjects touching the screen very hard with their knuckles, palm, fist or the back of their hand; these behaviours were recorded as responses only if the touches occurred within 3 cm of the onscreen image of the competitor. For example, if the competitor was visible in the centre of the screen and the subject banged their fist on the screen's far edge, this would not be counted. If there was uncertainty about whether a particular instance represented a directed response, we recorded it as a 0. We felt that these were conservative rules that would reduce the risk of recording as responses behaviours that were actually just expressions of frustration, not directed towards a competitor.

### (e) Data analysis

All quantitative analyses were carried out in R version 4.1.2 [37]. We used the package lme4 [38] to analyse data using a generalized linear mixed-effects model [39] with a binomial error function. The model included movement, features and trial number as fixed effects. Subject ID and competitor ID were included as random effects. We also analysed each subject's performance individually, again with lme4, using generalized linear models with binomial error functions. The models included features, movement and trial number as fixed effects. We used an *α* level of *p* < 0.05 for all analyses. More detail on data analysis is presented in the supplementary text.

## 3. Results

**Figure 3.**
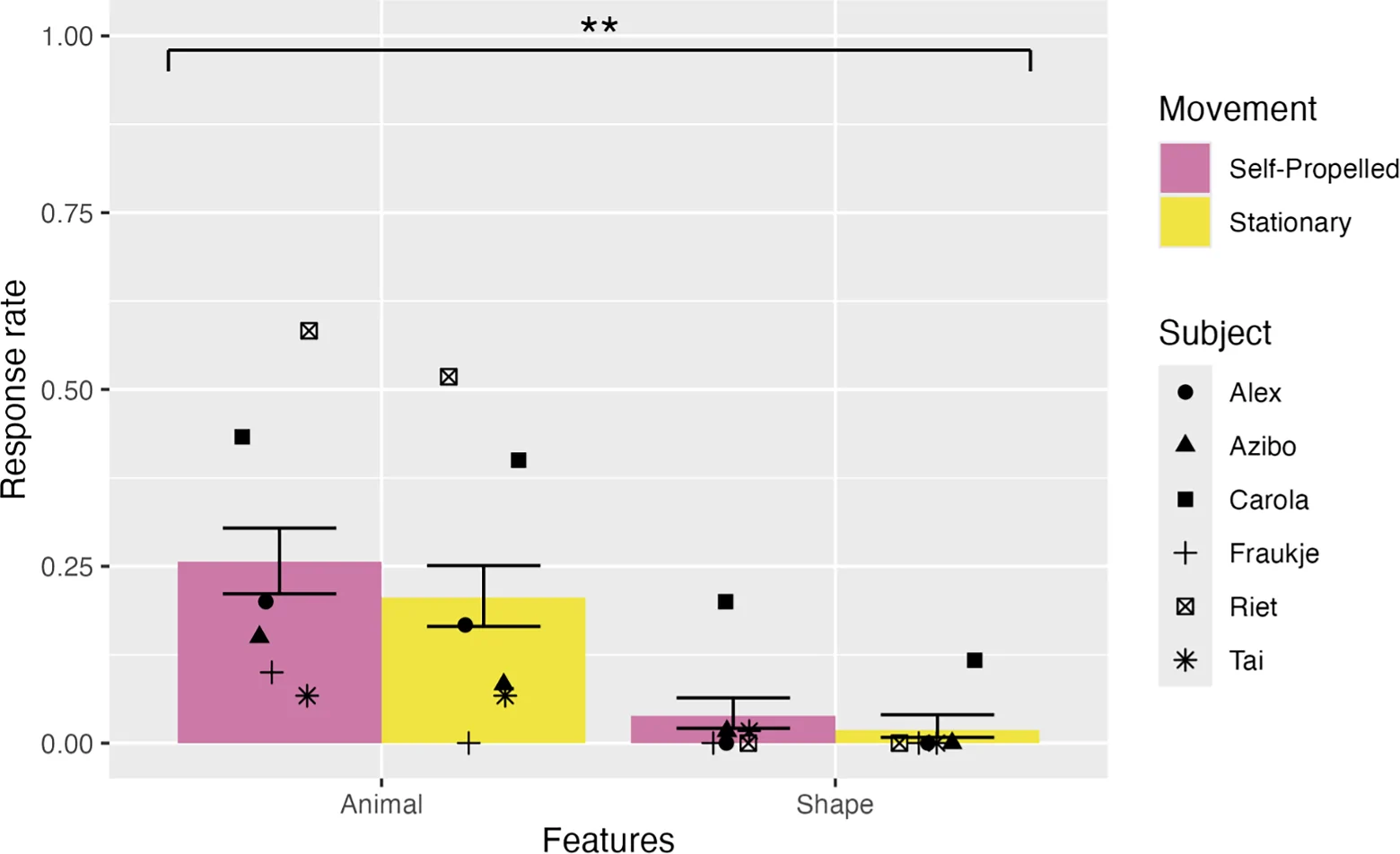
Bar graph showing group-level and individual proportion of trials with an emotive response across the four conditions. Points show individual mean response rates. Bars show group mean response rates. Error bars show exact binomial Clopper–Pearson 95% confidence intervals.

Figure 3 presents the proportion of responses we observed across all four conditions, both at the individual and group level. For the group-level data, the full-null model comparison was significant [$\chi^{2}$(3, *n* = 6) = 14.14, *p* = 0.003], so we continued with analysis of individual terms. We found that the chimpanzees responded significantly more frequently to animal competitors than to shapes [$\chi^{2}$(1, *n* = 6) = 8.38, *p* = 0.004]. The movement variable was marginally non-significant, indicating that subjects did not respond significantly differently to self-propelled and stationary competitors [$\chi^{2}$(1, *n* = 6) = 3.48, *p* = 0.06]. The likelihood of subjects responding to the competitors did not change over the course of the test [$\chi^{2}$(1, *n* = 6) = 0.71, *p* = 0.40]. Estimates, SEs, confidence intervals and odds ratios are presented in table S2 of the supplementary text. Table 2 shows the frequency of each behavioural response type. See the supplementary material for video clips of example responses.

**Table 2.** Overall response frequency according to type.

response type	frequency
vocalization	5.35%
facial expression	2.67%
gesture	15.51%
bang	83.96%
press	2.14%
jump	9.09%

*Note:* This table shows the frequency of different types of behavioural response as a proportion of the total number of trials featuring one or more responses. The reader should note that since more than one response type could be recorded for each trial, the percentages provided here sum to greater than 100%. Recall, however, that trials featuring more than one response type were, for the purposes of analysis, treated identically to trials in which only one response type was observed.

The full results of the individual-level analyses are presented in table S2 of the supplementary text. The features variable was a significant predictor of response proportion for five subjects, and was marginally non-significant for the final subject, Tai. The movement variable was significant for two subjects, Fraukje and Azibo. For Azibo, the trial variable was also significant: he responded more often as the experiment went on.

Although the individual subjects’ response frequencies varied significantly and were very low for some subjects, the overall pattern for every individual largely matched the whole-group pattern.

## 4. Discussion

In this study, chimpanzees were much more likely to display emotive responses towards animal-like competitors than towards geometric shapes; however, whether the competitor was self-propelled or stationary did not have a significant effect. Despite the differences in response levels across conditions being differences of degree, and not absolute—no subject responded to one type of competitor in *every* trial and to another type in *zero* trials—the pattern suggests that chimpanzees only viewed the animal-like competitors as intentional agents. Note, however, that although we did not find a significant effect for movement, we did find a trend; future studies should investigate this further.

The observed pattern of emotional responses indicates that they are not mere embodiments of chimpanzees’ frustration at losing out on rewards. If this were the case, we would expect equal response frequencies across all four conditions. Nevertheless, an argument could be made for the observed responses still being largely associated with frustration as opposed to showing that the chimpanzees were engaged in any agency attribution. For example, perhaps the subjects came to associate the feeling of frustration at losing a trial with the images of the virtual monkeys, such that they were driven to externalize their annoyance only when these two sets of stimuli co-occurred, and not when the monkey competitors were replaced by the geometric shapes.

This explanation is unlikely for two reasons. First, the emotional responses became evident in the first test session for three subjects, in the second test session for two subjects, and in the fourth test session for the final subject. This would represent a rapid associative period. Second, there were 24 occasions on which subjects responded emotionally towards the animal competitors in *motivational trials*. Sometimes subjects even went out of their way to approach the virtual monkeys and bang on their image/vocalize towards them before turning away and collecting the prey targets (see the supplementary material for video examples). By contrast, motivational trial responses to the shape competitors occurred only twice. This behaviour tells us two things. One, that the subjects’ emotional responses towards the competitors were decoupled from the simultaneous experience of frustration. It might nevertheless be argued that the subjects *remembered* the image of the monkeys as being associated with frustration from previous trials, and that this memory was enough to drive these responses in motivational trials. However, this explanation is insufficient to explain the fact that, two, the chimpanzees were sometimes willing to delay the capture of their target and the concomitant reward in favour of first approaching and interacting with the virtual monkeys. Such apparently deliberate behaviour seems incongruous with the idea of these responses being reflexive outpourings of frustration.

The current results are in line with those observed by Kano & Call [29]: in an anticipatory looking test, chimpanzees predicted the actions of a human hand shown in a video, but not a mechanical claw, despite both showing the same movement patterns. However, our results go beyond what was shown in that study in several important ways. First, although both the hand and the claw in [29] moved, they were not necessarily *self-propelled*: since the human arm and the arm of the mechanical claw extended out of frame, their motion was not obviously self-initiated; plausibly, another agent out of view was causing them to move (as was indeed the case with the mechanical claw). Experiments with human infants have shown that when they see a novel, featureless entity moving in a goal-directed manner, they interpret it as an agent; but when the same entity moves in the same way, but with a rod attached to it extending out of view, infants no longer see it as an agent—interpreting that something else was causing it to move and therefore it may not have been pursuing its own goals at all [40]. Plausibly, the same effect could have occurred in Kano & Call's study, meaning that the apes might not have interpreted the mechanical claw as self-propelled at all. This left us with an open question concerning how chimpanzees would interpret an entity that was, like the claw, featureless and not obviously goal-directed, but unlike the claw, clearly self-propelled. Our study answered that question: the moving competitors were unambiguously self-propelled, so chimpanzees’ lack of response to the moving shapes is a clearer signal than in [29].

Second, as mentioned in the Introduction, the entity with animal features in [29] was a human hand, not a new entity to the chimpanzees. These chimpanzees were accustomed to seeing a human hand grasping the object of their goal, but had never or rarely seen a plastic claw; a possible deflationary explanation of Kano & Call's results is that the chimpanzees were responding to a learned rule. In our study, by contrast, our subjects saw all competitors for the first time, so the fact that they responded more to competitors with animal features suggests that they categorize agents according to those characteristics.

Third, Kano & Call's [29] results provide no information concerning whether chimpanzees would interpret a hand (or another animal-featured entity) as an agent even if it did not move. Our study fills this gap, answering that indeed they do—since the difference in chimpanzees’ response rates to the stationary and moving animal competitors was not significant.

### (a) Parallels to humans’ attribution of agency

Research has shown that human children and adults attribute agency to novel entities using both behavioural and morphological diagnostic criteria. On the morphological front, humans tend to treat novel entities that possess *animal features,* such as body structure (a face and four limbs) or surface texture (skin and fur) as agents [7,41–43]. On the behavioural front, experimental evidence indicates that humans identify entities as agents if they display some evidence of *internal control* over their actions. Internal control can be demonstrated by the entity behaving in a variable way that is responsive to changes in the environment, or by its movement displaying equifinality, rationality and goal-directedness. For instance, if a novel entity is seen to select and move towards one of two targets [40,44,45], or deviate around an obstacle on its way to a target [46], or interact contingently with another entity—either through physical movement [47–50] or the exchange of signals [41,44,45,51,52]—humans will identify it as an agent. It is important to note here that, contrary to initial theories [2,53,54], self-propulsion alone is *not* sufficient to denote agency to human children or adults: when a novel entity moves on its own, but in a random manner that omits the above cues of rationality, goal-directedness, responsiveness to the environment or contingent interactions, humans do not treat it as an intentional agent [41,42,45–47,55–57]. But note that experiments also show that infants expect a novel entity to have innards when it is *both* agentive (i.e. has fur or interacts contingently with another agent) *and* self-propelled, but not when it is agentive and stationary [58,59], indicating that self-propulsion does play at least *some* role in young children's categorization of biological entities.

There are clear similarities between this description of humans’ agency attribution and the pattern of results we found in the current study for chimpanzees. Both species appear to use animal features as predictive cues of agency, and they do not interpret every object that moves on its own as an agent. Therefore, our findings may suggest that chimpanzees and humans use some of the same rules to rapidly categorize and discriminate agents and physical objects.

### (b) Implications

This interpretation of our findings implies that categorizing agency using features and not relying on self-propelled movement alone is a conserved predisposition, more evolutionarily ancient than our split from chimpanzees. It is feasible that the trait is older still, and is also present in other great ape species or even more distantly related animals, having arisen in response to general challenges faced by a number of species. Because agents, by definition [1], have goals and can perceive and react to the environment, and thus must have psychological states that can be reasoned about, the ability to construe certain entities as agents may be used by an animal to anticipate the behaviour of their prey, their predators or their conspecifics. Such predictions are useful in enabling an animal to avoid deleterious actions (though, to be clear, we do not mean to suggest that agency attribution and psychological reasoning are the *only* means by which such action predictions can be arrived at; simpler, perceptual or rule-based mechanisms may play this role for many species).

To date, there is little experimental or theoretical literature considering agency attribution in non-human species; most work on agency considers only humans, and even within this field, there has been little discussion of the possible reasons for, and circumstances of, its evolution. The results of the current study suggest that the question of which species attribute agency, why and how, merits further investigation; below, we delineate a selection of potential investigative paths.

Separately, our results have implications for VEs as an experimental paradigm. That chimpanzees spontaneously treat virtual animals as agents is important and heightens the relevance of this method because it means that they do not simply view these stimuli as images on a screen, devoid of any social connections to the real world; rather, chimpanzees clearly imbue these animated entities with some referential value. This supports the validity of using VEs to investigate other aspects of chimpanzees’ social cognition.

### (c) Limitations and future directions

Although self-propulsion was common to both the animal and shape competitors in this study, the ways in which the types of competitor achieved this self-propulsion were different: the shapes slid rigidly across the virtual space, while the animals walked with their legs. Evidence indicates that human infants are more likely to attribute agency to novel entities showing non-rigid, irregular, rhythmic ‘biological motion’ in comparison to novel entities that move rigidly and without deviating [60,61] and that other taxa, including cats and pigeons, are sensitive to biological motion when shown via point-light displays [62–64] (but see [65,66]). It is possible, therefore, that subjects were less likely to denote the shape competitors in our study as agentive because they moved non-biomechanically. Future studies could endow the shape competitors with a more ‘animal-like’ motion pattern, such as hopping, to match the animal competitors—or, conversely, make the animal competitors glide along without using their legs.

Another limitation was that the behavioural coding was partially subjective. Although the interrater agreement was substantial, it was lower than is common for experiments involving coding of more clear-cut behaviours (such as pointing in forced-choice paradigms). One source of potential ambiguity arose from subjects differing in their general interaction patterns with the VE; this meant that coders had to take into account, for example, the force with which a subject generally touched the screen when deciding whether a touch was to be counted as a ‘bang’. Also, to decide in ambiguous cases whether a touch was within 3 cm of the onscreen competitor, coders had to measure the distance as captured on the video and from this calculate the real-life distance, which was something of a blunt instrument.

A fruitful avenue of research would be to run similar tests on other great ape species. If feature-based agency attribution is truly a homologous trait between chimpanzees and humans, we would expect it to work similarly in the bonobos (*Pan paniscus*), with whom we are equally closely related. If bonobos do display a matching pattern, we could then extend the question to the other great apes, gorillas (*Gorilla gorilla*) and orangutans (*Pongo spp*), and thereby attempt a taxonomic reconstruction of the evolution of our agency attribution skills. Although the question of how other great apes categorize agents and non-agents has not been directly investigated, one set of studies investigating chimpanzees’, bonobos’ and orangutans’ understanding of false beliefs found evidence consistent with the current results: namely, that all three species anticipated the behaviour of a human actor but not of coloured geometric shapes [13,67]. Also, when shown novel geometric shapes that possessed both morphological and behavioural agency cues, bonobos discriminated agents on the basis of their third-party interactions and preferred hinderers over helpers [68].

Perhaps even more interesting would be to investigate whether more distant species, for which the challenges of anticipating others’ behaviour are equally relevant, also show evidence of discriminating intentional agents from non-agentive novel entities; and if so, whether they use any of the same diagnostic characteristics that humans, and now chimpanzees, have been shown to use. Taxa that have already been shown to be capable of complex cognition, such as corvids, cetaceans and domestic dogs, could represent viable study subjects. Direct comparison across species would be useful: testing both humans and non-human animals with the same methods is the only way to ensure that methodological artefacts do not skew our perceptions one way or another. Further, if a comparison using a more traditional paradigm, such as gaze-following or violation-of-expectation, supported the results of the current study, it would validate our novel methodology.

It would also be beneficial to identify *which* specific physical features chimpanzees use to identify agents. The animal-like competitors in the current study possessed several morphological features associated with agency, most prominently a face and four limbs; a follow-up experiment could test whether chimpanzees would respond in the same way to, for example, an object with no limbs but with a face, as human infants do [41].

Another aspect that remains yet to be evaluated is whether chimpanzees, like humans, identify entities as agents if they hold internal control over their own actions—that is, if the entities can select their own goals and the actions they use to achieve them, or react to dynamic changes in the world around them. Future experiments could seek to elucidate this by using competitors that lack animal features but engage in reciprocal, real-time communication with other agents or change their behaviour in response to environmental changes, taking cues from prior research with human children. For example, researchers could adapt the method used in [57], with a featureless box that shows goal-directedness and efficiency through more complex movement patterns, such as approaching the same target in either a straight line or a detour path, depending on whether an obstacle is present. Or, following [45], subjects could be tasked with evaluating the agency of a novel object that produces sounds to engage in a back-and-forth conversation with an experimenter. Generating detailed, systematic evidence on the specific behavioural and/or featural factors that lead chimpanzees to categorize entities as agentive could allow us to make some initial speculations regarding the selective pressures governing the trait's evolution.

In conclusion, the outcomes of this study provide evidence in support of the idea that chimpanzees use animal features, but not necessarily simple self-propulsion cues, to identify agents. This pattern parallels that seen in humans, raising the possibility that feature-based agency attribution is a homologous trait between our two species. We suggest further experiments to fortify this contention, including extending the question to other species and comparing different relevant attributes to refine our understanding of human and non-human agency attribution.

## Supplementary Material



## Data Availability

Data can be accessed at OSF [69]. Supplementary material is available online [70].
