# Supplementary material for: Chimpanzees attribute agency to virtual animals, but not to self-propelled shapes
Source: Proc Biol Sci. 2026 Sep 9;293(2078):20261574. doi: 10.1098/rspb.2026.1574 (PMC13561048; doi:10.1098/rspb.2026.1574)
Supplement: Supplementary file 1 [file rspb.2026.1574.zip › rspb.2026.1574/rspb20261574_si_001.docx]

**Chimpanzees attribute agency to virtual animals, but not to self-propelled shapes**

Emilie Rapport Munro, Matthias Allritz, Kenneth Schweller, Francine L. Dolins, Daniel B. M. Haun, & Josep Call. Proceedings of the Royal Society B; article DOI: 10.1098/rspb

**Supplementary Text**

**Subjects and Housing**

Chimpanzees at the Wolfgang Köhler Primate Research Center live in social settings; one subject (Alex) is part of a small group of 6 chimpanzees, of which he is the only male, while the other five are part of a larger group of over 20 individuals. Chimpanzees at the WKPRC have access to extensive, multilevel indoor enclosures with seasonal access outdoors, and are provided with foraging boxes and varied enrichment food items.

Individuals were separated from the rest of the group during research (except for mothers and dependent infants), with each subject participating for no more than 15 minutes per day. Subjects were never food- or water-deprived. If an individual showed signs of wanting to end participation before completing their research for the day (e.g., by going to the door or refusing to touch the screen), they were let out to rejoin the group. Although the nature of the study implied that we expected some responses indicative of displeasure from the subjects, the acceptable responses were within strict limits that were agreed with the keepers before the start of data collection. Clear ethical protocols were in place for the eventuality that an individual might display behaviours outside of acceptable bounds (such as self-scratching, fear-grinning, or screaming): testing would be immediately discontinued with that individual and only recommenced subject to review and approval from the head keeper.

All animal husbandry and scientific research conducted at WKPRC complies with the EAZA Minimum Standards for the Accommodation and Care of Animals in Zoos and Aquaria and the WAZA Ethical Guidelines for the Conduct of Research on Animals by Zoos and Aquariums. Ethical approval for the study was granted by the Ethics Committee of the School of Psychology and Neuroscience, University of St Andrews, and by the ethical committee of the WKPRC (consisting of the head keeper, zoo veterinarian, and research coordinator).

**Table S1**

*Example trial order within a session*

| Trial name | Trial number | Competitor feature | Competitor movement | Trial type | Mini-block |
| --- | --- | --- | --- | --- | --- |
| BlueCube_Catch_Boar | 1 | Shape | Self-Propelled | Test | 1 |
| Rhesus_Ignore_Pig | 2 | Animal | Stationary | Motivation | 1 |
| Rhesus_Ignore_Pig | 3 | Animal | Stationary | Motivation | 1 |
| BlueCube_Catch_Boar | 4 | Shape | Self-Propelled | Test | 1 |
| BlueCube_Catch_Boar | 5 | Shape | Self-Propelled | Test | 1 |
| Rhesus_Ignore_Pig | 6 | Animal | Stationary | Motivation | 1 |
| BlueCube_Away_Boar | 7 | Shape | Self-Propelled | Motivation | 2 |
| Rhesus_Stationary_Boar | 8 | Animal | Stationary | Test | 2 |
| BlueCube_Away_Boar | 9 | Shape | Self-Propelled | Motivation | 2 |
| BlueCube_Away_Boar | 10 | Shape | Self-Propelled | Motivation | 2 |
| Rhesus_Stationary_Boar | 11 | Animal | Stationary | Test | 2 |
| Rhesus_Stationary_Boar | 12 | Animal | Stationary | Test | 2 |
| Capuchin_Away_Pig | 13 | Animal | Self-Propelled | Motivation | 3 |
| RedPyramid_Stationary_Pig | 14 | Shape | Stationary | Test | 3 |
| RedPyramid_Stationary_Pig | 15 | Shape | Stationary | Test | 3 |
| Capuchin_Away_Pig | 16 | Animal | Self-Propelled | Motivation | 3 |
| Capuchin_Away_Pig | 17 | Animal | Self-Propelled | Motivation | 3 |
| RedPyramid_Stationary_Pig | 18 | Shape | Stationary | Test | 3 |
| Capuchin_Catch_Boar | 19 | Animal | Self-Propelled | Test | 4 |
| Capuchin_Catch_Boar | 20 | Animal | Self-Propelled | Test | 4 |
| RedPyramid_Ignore_Boar | 21 | Shape | Stationary | Motivation | 4 |
| RedPyramid_Ignore_Boar | 22 | Shape | Stationary | Motivation | 4 |
| RedPyramid_Ignore_Boar | 23 | Shape | Stationary | Motivation | 4 |
| Capuchin_Catch_Boar | 24 | Animal | Self-Propelled | Test | 4 |

**Power Analysis**

We assumed a data-generating process in which the predicted probability of a positive response in a given trial was given as

probability(response) = plogis( intercept + slope_movement*movement
+ slope_features*features )

Here, *plogis* refers to the inverse logit function (translating from log odds to probability space). The *intercept* term refers to the baseline response rate when agents are neither moving nor have animal features. *Movement* and *features* are binary variables indicating whether in a given trial the agent was moving or not, and whether it had animal features or not, respectively. Finally, *slope_movement* and *slope_features* refer to the expected increase in the response if *movement* or *features*, respectively, was set to 1 rather than 0 in a given trial. To obtain actual estimates for the intercept and slopes, we built upon our previous work. In the previous study that inspired this one [31], we saw emotional responses towards competitors in 34.3% of trials. The competitors in that experiment were moving animals. We therefore assumed that responses in our new experiment would occur when moving animal competitors were present, with the same probability of 0.343. Conversely, we assumed that the baseline response, when the competitor was neither moving nor had animal features, would be close to zero. Here, we set a speculative intercept of 0.05. Regarding the assumed size of the two slope terms, because no data were available to us a priori to estimate the relative contributions of self-propulsion and animal features to the outcome, we set both predictors to equal values. The slope for the *movement* variable and for the *features* variable were therefore each set, in link space, to half the difference between the maximum response (0.343) and intercept (0.05). Simulated trial outcomes were then generated by drawing samples from a binomial distribution that used this initial, formula-based prediction as the probability of showing a response.

We then generated 2000 independent sets of simulated data, completing this process once each with 144 trials per simulation (36 trials per cell of the 2 × 2 design), once with 240 trials (60 trials each per cell), and once with 300 trials (75 trials each per cell). We analysed each dataset using a binomial generalised linear model with response ~ movement + features (using the function glm from the R package lme4 for model fitting and the function drop1 for estimating *p*-values). We aimed to optimise for a situation in which >80% of the 2000 simulations showed a significant effect (*p*<.05) for both features and movement.

**Data analysis**

For the group-level analysis, we initially fitted a maximal model that included not only the random intercept for subject ID but also random slopes for features and movement. This model failed to converge, even after increasing the maximum number of function evaluations it should try. Next, we tried removing each random slope one at a time; we found a working model retaining the random slope of features but lacking the random slope of movement [1]. This was the model we used for analysis, having ascertained that it met the assumptions of a GLMM (uniformity in quantile-quantile and residual plots, and absence of multi-collinearity and zero-inflation).

We used likelihood ratio tests to ascertain the significance of each full model by comparing it to a null model (specified as response ~ 1 plus the same random effect structure as in the full model). If the full model was significant, we used the drop1() function to carry out likelihood ratio tests to assess the significance of individual predictors. We extracted parameter estimates and standard errors from the model summary and used them to compute odds ratios and confidence intervals.

When we carried out the individual-level analyses, for three subjects (Alex, Fraukje and Riet) initial GLM model estimates showed inflated standard errors due to complete separation in the data. To combat this, we analysed these subjects’ data using Firth logistic regression with the R package logistf. This method uses penalised likelihood estimation, making it less biased and more robust to complete separation issues [2].

**Results**

**Table S2**

*Group-level and individual-level analysis results*

| Subject | Term | Estimate | SE | OR | 95% CI | Likelihood Ratio Test | | |
| --- | --- | --- | --- | --- | --- | --- | --- | --- |
|  |  |  |  |  |  | df | χ^2^ | p |
| Group | Features | -4.01** | 1.21 | 0.02 | [0.002, 0.20] | 1 | 8.38 | .004 |
|  | Movement | -0.44 | 0.34 | 0.66 | [0.33, 1.26] | 1 | 3.48 | .06 |
|  | Trial | -0.08 | 0.09 | 0.92 | [0.77, 1.11] | 1 | 0.71 | .40 |
| Alex | Features | -3.95** | 1.38 | 0.02 | [0.0002, 0.14] | 1 | 27.31 | 1.74e^-07^ |
|  | Movement | -0.21 | 0.52 | 0.80 | [0.27, 2.34] | 1 | 0.16 | .69 |
|  | Trial | 0.04 | 0.27 | 1.00 | [0.59, 1.77] | 1 | 0.0002 | .99 |
| Azibo | Features | -2.28** | 1.06 | 0.10 | [0.006, 0.56] | 1 | 7.83 | .005 |
|  | Movement | -1.23* | 0.61 | 0.29 | [0.08, 0.93] | 1 | 4.33 | .04 |
|  | Trial | 0.84** | 0.35 | 2.32 | [1.22, 4.90] | 1 | 6.73 | .009 |
| Carola | Features | -1.43** | 0.32 | 0.24 | [0.13, 0.44] | 1 | 22.1 | 2.63e^-06^ |
|  | Movement | -0.27 | 0.30 | 0.76 | [0.42, 1.37] | 1 | 0.82 | .37 |
|  | Trial | -0.26 | 0.15 | 0.77 | [0.57, 1.03] | 1 | 2.91 | .09 |
| Fraukje | Features | -2.66** | 1.39 | 0.07 | [0.0005, 0.61] | 1 | 6.42 | .01 |
|  | Movement | -3.02* | 1.53 | 0.05 | [0.0003, 0.71] | 1 | 4.98 | .03 |
|  | Trial | 0.23 | 0.43 | 1.23 | [0.47, 3.09] | 1 | 0.24 | .62 |
| Riet | Features | -5.84** | 1.40 | 0.003 | [2.26e^-05^, 0.02] | 1 | 115 | 0 |
|  | Movement | 0.36 | 0.48 | 1.44 | [0.55, 3.86] | 1 | 0.56 | .46 |
|  | Trial | -0.42 | 0.22 | 0.65 | [0.42, 1.00] | 1 | 3.91 | .05 |
| Tai | Features | -1.80 | 1.10 | 0.17 | [0.009, 1.02] | 1 | 3.73 | .05 |
|  | Movement | -0.16 | 0.70 | 0.85 | [0.20, 3.39] | 1 | 0.05 | .82 |
|  | Trial | 0.56 | 0.40 | 1.76 | [0.86, 4.25] | 1 | 2.32 | .13 |

*Note.* Asterisks denote significant effects*.* For estimates, the reference level for Features was Animal, and the reference level for Movement was Self-Propelled. 95% confidence intervals were computed using the profile likelihood method for individual subjects and the Wald method for the group, and are presented on the odds ratio (exponentiated) scale.

**References**

1. Barr D, Levy R, Scheepers C, Tily H. 2013 Random effects structure for confirmatory hypothesis testing: Keep it maximal. J. *Memory and Language* 68(3). <https://doi.org/10.1016/j.jml.2012.11.001>
2. Heinze G, Schemper M. 2002 A solution to the problem of separation in logistic regression. *Statistics in Med*. 21(16), 2409–2419. https://doi.org/10.1002/sim.1047

**Electronic Supplementary Materials legends**

**Video A**

*Video clips of example responses in Test trials*

*Note.* This video shows various subjects displaying emotional responses towards different competitor types in test trials. The type of response is labelled for each clip.

**Video B**

*Video clips of example responses in Motivation trials*

*Note.* This video shows various subjects displaying emotional responses towards different competitor types in motivational trials in the test phase. The type of response is labelled for each clip.
